# Etiology and Clinical Characteristics of Severe Pneumonia Among Young Children in Thailand: Pneumonia Etiology Research for Child Health (PERCH) Case–Control Study Findings, 2012–2013

**DOI:** 10.1097/INF.0000000000002768

**Published:** 2021-08-25

**Authors:** Charatdao Bunthi, Julia Rhodes, Somsak Thamthitiwat, Melissa M. Higdon, Somchai Chuananon, Tussanee Amorninthapichet, Wantana Paveenkittiporn, Malinee Chittaganpitch, Pongpun Sawatwong, Laura L. Hammitt, Daniel R. Feikin, David R. Murdoch, Maria Deloria-Knoll, Katherine L. O’Brien, Christine Prosperi, Susan A. Maloney, Henry C. Baggett, Pasakorn Akarasewi

**Affiliations:** From the * Division of Global Health Protection, Thailand Ministry of Public Health–US Centers for Disease Control and Prevention Collaboration, Nonthaburi, Thailand; † Department of International Health, International Vaccine Access Center, Johns Hopkins Bloomberg School of Public Health, Baltimore, Maryland; ‡ Nongbualamphu Hospital, Nongbualamphu; § Sakaeo Crown Prince Hospital, Sakaeo, Thailand; ¶ National Institute of Health, Ministry of Public Health, Nonthaburi, Thailand; ‖ Department of Pathology, University of Otago, Christchurch, New Zealand; Microbiology Unit, Canterbury Health Laboratories, Christchurch, New Zealand; ** Division of Global HIV and TB, Center for Global Health, Centers for Disease Control and Prevention, Atlanta, GA; †† Division of Global Health Protection, Centers for Disease Control and Prevention, Atlanta, GA; ‡‡ Department of Disease Control, Ministry of Public Health, Nonthaburi, Thailand.

**Keywords:** Thailand, pneumonia, etiology, childhood, PERCH

## Abstract

Pneumonia remains the leading cause of death among children <5 years of age beyond the neonatal period in Thailand. Using data from the Pneumonia Etiology Research for Child Health (PERCH) Study, we provide a detailed description of pneumonia cases and etiology in Thailand to inform local treatment and prevention strategies in this age group.

**Methods:**

PERCH, a multi-country case–control study, evaluated the etiology of hospitalized cases of severe and very severe pneumonia among children 1–59 months of age. The Thailand site enrolled children for 24 consecutive months during January 2012–February 2014 with staggered start dates in 2 provinces. Cases were children hospitalized with pre-2013 WHO-defined severe or very severe pneumonia. Community controls were randomly selected from health services registries in each province. Analyses were restricted to HIV-negative cases and controls. We calculated adjusted odds ratios (ORs) and 95% CIs comparing organism prevalence detected by nasopharyngeal/oropharyngeal (NP/OP) polymerase chain reaction between cases and controls. The PERCH Integrated Analysis (PIA) used Bayesian latent variable analysis to estimate pathogen-specific etiologic fractions and 95% credible intervals.

**Results:**

Over 96% of both cases (n = 223) and controls (n = 659) had at least 1 organism detected; multiple organisms were detected in 86% of cases and 88% of controls. Among 98 chest Radiograph positive (CXR+) cases, respiratory syncytial virus (RSV) had the highest NP/OP prevalence (22.9%) and the strongest association with case status (OR 20.5; 95% CI: 10.2, 41.3) and accounted for 34.6% of the total etiologic fraction. Tuberculosis (TB) accounted for 10% (95% CrI: 1.6–26%) of the etiologic fraction among CXR+ cases.

**Discussion:**

More than one-third of hospitalized cases of severe and very severe CXR+ pneumonia among children 1–59 months of age in Thailand were attributable to RSV. TB accounted for 10% of cases, supporting evaluation for TB among children hospitalized with pneumonia in high-burden settings. Similarities in pneumonia etiology in Thailand and other PERCH sites suggest that global control strategies based on PERCH study findings are relevant to Thailand and similar settings.

## Abstract 2

Supplemental Digital Content is available in the text.

Pneumonia remains the leading cause of death among children <5 years of age beyond the neonatal period,^1–3^ accounting for 15.3% of deaths globally and 9.0% in Thailand in 2012.^4^ Our understanding of childhood pneumonia etiology in the developing world largely comes from research studies in the 1970s through the 1990s.^5,6^ To estimate pneumonia burden and etiology, the Thailand Ministry of Public Health (MoPH) and United States Centers for Disease Control and Prevention conducted surveillance for acute lower respiratory tract infection (ALRI) among hospitalized cases in 2 provinces in Thailand from 2003 to 2014.^7^ From this surveillance among childhood ALRI cases, we estimated the prevalence of pathogens such as influenza (8.2%^8^) and respiratory syncytial virus (RSV) (19.5%^9,10^). However, these reports were based on single tests like blood culture or polymerase chain reaction (PCR) on upper airway specimens which may lack sensitivity and specificity for bacteria. Analyses of data from this surveillance system did not take into account assay sensitivity and specificity or integrate the results from multiple tests in individual cases.^11^ This classical analytic approach required assignment of etiology based on expert opinion when multiple tests were positive and precluded development of a probability-based etiology distribution, either at individual or population level. This surveillance system also did not apply World Health Organization (WHO) case definitions for childhood pneumonia limiting comparisons to other childhood pneumonia studies. The Pneumonia Etiology Research for Child Health (PERCH) Study offered an important opportunity to address these limitations and deepen our understanding of severe childhood pneumonia in Thailand and the region by employing highly standardized clinical and laboratory methods^12^ and novel analytic approaches.

The PERCH Study was a multisite, case–control study of hospitalized children 1–59 months of age with severe and very severe pneumonia conducted at 9 sites in 7 countries, including 2 sites in Thailand.^13–15^ PERCH sought to advance our understanding of childhood pneumonia etiology in the context of economic development, improvements in health status, and pneumonia-specific interventions^16^; therefore, it included sites that would collectively be representative of the future epidemiologic environments. The Thailand site represents a setting expected to become more common as more countries advance in their development. Our study is a Thailand-specific analysis that provides a more detailed description of the pneumonia cases and etiology beyond the overall PERCH study findings^15^ that informs and has implications for local treatment and prevention strategies.

## METHODS

### Setting

Thailand is an upper middle-income country with higher gross national income per capita^17^ than most other PERCH sites,^13^ although incomes in the study provinces are lower than incomes of Thailand overall; 2012 GDPs were approximately US $2000 in study provinces compared with >US $14,000 in Bangkok.^18^ Thailand’s health status indicators were better than other PERCH sites, including lower under 5 mortality (13.90/1000 live births)^19^ and lower HIV incidence [0.16 (0.15–0.18) per 1000 population all ages in 2012^20^], achieved in part through a successful program for prevention of mother-to-child HIV transmission, which has virtually eliminated HIV among infants.^21^ Primary health care services, including vaccination, are free for all Thais.^22^ Thailand’s National Vaccination Program did not include pneumococcal conjugate vaccine (PCV) or *Haemophilus influenzae* type b (Hib) conjugate vaccine during the study period. In 2010, Thailand’s Advisory Committee on Immunization Practice expanded its influenza vaccination recommendations to include children 6-months-old to 2-years-old^23^ and those with chronic illness, although vaccine uptake in young children has been limited.^24^ PERCH enrollment in Thailand was conducted from 2012 to 2014 at Sa Kaeo Crown Prince Hospital (382 beds) and Nakhon Phanom Hospital (356 beds), each serving as a referral hospital for its province. Sa Kaeo and Nakhon Phanom border Cambodia and Lao PDR, respectively, have approximately 40,000 children <5-years-old each and consist of largely rural populations.

### Selection of Participants

We defined cases as children 1–59 months of age hospitalized with pre-2013 WHO defined severe or very severe pneumonia.^25,26^ Severe pneumonia was defined as having cough or difficulty breathing and lower chest wall indrawing; very severe pneumonia was defined as cough or difficulty breathing and at least one of the following: central cyanosis, difficulty breastfeeding/drinking, vomiting everything, convulsions, lethargy, unconsciousness or head nodding. Cases were excluded for hospitalization within the previous 14 days, having been discharged as a PERCH case within the past 30 days, not residing in the study catchment area or resolution of lower chest wall indrawing following bronchodilator therapy for those with wheeze. PERCH research nurses trained on standardized clinical assessments^27^ performed case screening 24 hours per day/7 days per week. Each site enrolled for 24 consecutive months: January 2012–December 2013 (Nakhon Phanom), March 2012–February 2014 (Sa Kaeo).

Community controls were randomly selected from comprehensive lists of children 1–59 months of age drawn from health services registries, which include virtually all children in each province. Controls were enrolled year-round and frequency matched to the age-group distribution of the cases on a monthly basis. We enrolled controls regardless of respiratory symptoms but excluded children with severe or very severe pneumonia; we chose not to exclude children with respiratory symptoms to have controls that are most representative of the general population and the least subject to selection bias.^28^ We aimed to enroll 38 controls monthly (25 in Nakhon Phanom and 13 in Sa Kaeo) to ensure at least a 1.5:1 control:case ratio based on projected case enrollment.

### Clinical Procedures

PERCH study staff at all sites underwent a standardized training in the clinical assessment of the child with cough or difficulty breathing, techniques for collection of NP/OP swabs and anthropometry.^27^ Cases underwent a standardized clinical examination at admission and at 24 and at 48 hours. Controls were similarly assessed at enrollment. Malnutrition was defined as WHO weight-for-age Z score < −3 (severe) and ≥ −3 to < −2 (moderate).^29^ For cases, a follow-up visit or phone interview was conducted 30 days postadmission (within a window of 21–90 days) to determine vital status.

Chest Radiographs (CXRs) were obtained from cases at enrollment and interpreted by 2 members of a panel of radiologists and pediatricians trained in the WHO method^30,31^ and blinded to site and clinical factors. CXRs were classified as consolidation, other infiltrate, both, normal or uninterpretable; children with consolidation, other infiltrate or both were defined as having CXR+ pneumonia.

### Sample Collection and Laboratory Testing

Specimen collection procedures, laboratory testing methods and determination of pathogen-specific density thresholds have been described separately.^32–37^ In brief, blood and nasopharyngeal/oropharyngeal (NP/OP) specimens were collected from cases and controls. A 33-pathogen multiplex quantitative PCR (FTD Resp-33, Fast Track Diagnostics, Sliema, Malta) and cultures were used to test NP/OP swabs (of cases and controls; cultures were done for *Streptococcus pneumoniae* alone). Blood samples were tested for *S. pneumoniae* by PCR for cases and controls and cultures were performed for cases only. All cases were tested for HIV by serum antibody assay, followed by PCR testing for antibody-positive cases <18 months of age. Due to the low prevalence of HIV in Thailand, controls were not tested for HIV. A single-induced sputum specimen was obtained from each case for *Mycobacterium tuberculosis* (MTB) culture.^12^ Antibiotic use before specimen collection was determined by serum antibiotic activity,^38^ by documented administration at study hospital or elsewhere, and by parental report.

In general, pathogen positivity was determined according to the presence or absence of the pathogen, but for 4 pathogens with similar prevalence in cases and controls, positivity was defined using quantitative PCR density thresholds; these included *S. pneumoniae* (≥2.2 log_10_ copies/mL) from whole blood^34,35,37^ and *S. pneumoniae* (≥6.9 log_10_ copies/mL),^35,37^ *H. influenzae* (≥5.9 log_10_ copies/mL),^34^ cytomegalovirus (CMV, ≥4.9 log_10_ copies/mL), *Pneumocystis jirovecii* (≥4 log_10_ copies/mL)^34^ from NP/OP and (CMV threshold analysis available from authors).^34,35,37^

### Statistical Analysis

We used logistic regression to calculate age-adjusted odds ratios (ORs) and 95% CIs comparing demographic and environmental characteristics as well as the prevalence of organisms detected by NP/OP PCR between cases and controls. ORs for the NP/OP prevalence of individual pathogens were also adjusted for the presence of the other pathogens detected (ie, to compare persons who are otherwise similar with respect to the other measured pathogens) because pathogens can be associated with each other and the presence of one can be influenced by the presence/absence of another.

We also present results from the PERCH Integrated Analysis (PIA), the methods of which are described in detail elsewhere.^39^ In brief, the PIA uses Bayesian latent variable analysis to estimate the etiologic distribution of pneumonia and the etiologic probability distribution of each individual case. The PIA integrates the results of blood cultures, pneumococcal whole blood PCR, TB tests and NP/OP PCR, while accounting for the specimen- and test-specific sensitivities and specificities and for the reduced sensitivity due to prior antibiotic use.^40,41^ In the PERCH study, the specificity of blood cultures and TB were assumed to be 100% and their sensitivity estimates were specified as a range. The sensitivity of TB was assumed to be 10%–30% and the sensitivity for blood culture for all other bacteria ranged from 5% to 50% (see reference ^15^, Appendix Section III.B.6). The specificity of NP/OP PCR was estimated in the PIA model using the control prevalence; the sensitivity prior specified for all NP/OP PCR pathogens was the range 50%–90%, except for *Salmonella* species and *Legionella* species, which were 0.5%–90%. The sensitivity of whole-blood pneumococcal PCR was assumed to be 12%–65%.

For every case, each pneumonia pathogen was assigned a probability for being the cause of the severe/very severe pneumonia episode with all probabilities adding to 1. The population-level estimate for each pathogen is the average of the individual case probabilities and has a 95% credible interval (95% CrI), the Bayesian analogue of the CI. The PIA assumes that a pneumonia event is caused by a single pathogen. For cases with multiple pathogens detected in silver standard measurements (blood cultures, lung aspirates or pleural fluid), the model cannot distinguish which one is the dominant cause and distributes the etiology probability equally across the pathogens detected. For cases who are negative by silver standard measurements, the cause is distributed across multiple pathogens according to the strength of evidence for each pathogen from other measurements (whether the pathogen was detected in multiple samples from the individual, the pathogen’s prevalence among cases, the pathogen’s OR and a priori assumptions regarding sensitivity and etiology).

Analyses were performed using SAS 9.4 (SAS Institute, Cary, NC), R Statistical Software (The R Development Core Team, Vienna, Austria) and BAKER for the PIA analysis.^39^

### Ethical Considerations

Parents or legal guardians of participants provided written informed consent in Thai. The study was approved by (1) the Thailand Ministry of Public Health Ethical Review Committee, (2) the Institutional Review Board of the Johns Hopkins Bloomberg School of Public Health and (3) an Institutional Review Board of the US Centers for Disease Control and Prevention (Protocol 6076).

## RESULTS

### Case and Control Enrollment

**FIGURE 1.**
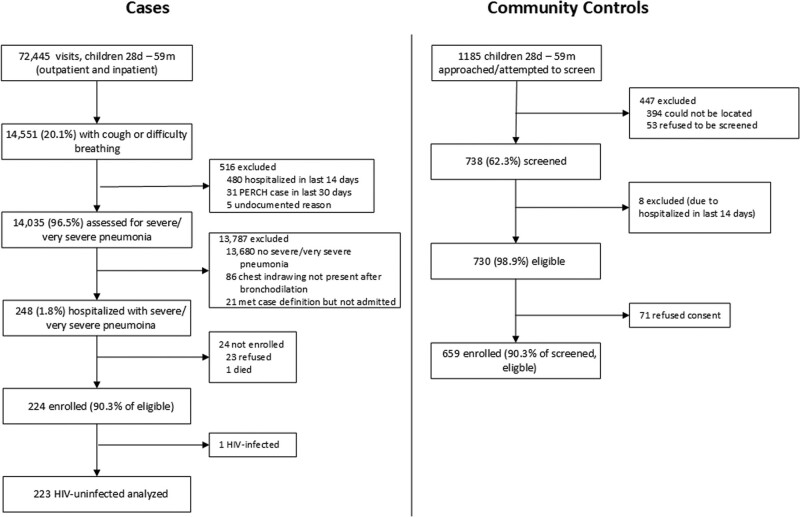
Case and control (1–59 months) enrollment flow—PERCH, Thailand, 2012–2013. *HIV testing was not performed on controls. ^1^Description of symbols: Lines represent the 95% credible interval for the etiologic fraction. The size of the symbol is scaled based on the ratio of the estimated etiologic fraction to its standard error. For two identical etiologic fraction estimates, the estimate with a larger symbol is more informed by the data. ^2^Chest radiograph positives defined as consolidation, other infiltrate, or both. pna, pneumonia; PERCH; Pneumonia Etiology Research for Child Health; HIV, human deficiency virus.

We screened 14,551 children <5 years of age with cough or difficulty breathing (Fig. 1). Of 248 eligible patients, 23 (9.3%) refused enrollment, 1 died before consent could be obtained and 224 (90.3%) were enrolled. Among 224 enrolled cases, 147 were from Nakhon Phanom and 77 from Sa Kaeo. One case was HIV-positive; subsequent analyses were limited to HIV-negative cases. Of 1185 randomly selected potential controls, 738 (62.3%) were screened; of these, 98.1% were eligible, of whom 90.3% (n = 659) enrolled (Fig. 1).

### Case and Control Characteristics

Among 223 HIV-negative PERCH cases, 98 (43.8%) had radiographically confirmed pneumonia (CXR+) (Table 1). Over half (53.3%) of all cases were between 6- and 23-months-old. Case households had lower monthly income than control households (*P* = 0.003), but we found no substantial differences in household size or maternal education. Cases were less likely than controls to be fully vaccinated for age for DTP (diphtheria, pertussis and tetanus) vaccine (*P* = 0.001). Only 1 of 182 cases and 2 of 442 controls that met Thailand’s recommended criteria for influenza vaccination were vaccinated (*P* = 0.99). Only 2 cases and no controls received one or more doses of PCV (*P* = 0.98, not shown); 9 (4.2%) cases and 19 (2.9%) controls had received one or more doses of Hib vaccine (*P* = 0.39, not shown). Malnutrition (WHO weight-for-age Z-score lower than −2), was more common among all cases (27.0%) compared with controls (7.0%, *P* value <0.001)

**TABLE 1.** Demographic and Clinical Characteristics of PERCH Cases and Controls—PERCH, Thailand, 2012–2013

	All Cases, n (%)	CXR+ Cases^a^n (%)	Controlsn (%)	All Cases vs. Controls, *P* Value^b^	CXR+ Cases vs. Controls, *P* Value^b^
**All**	223	98	659		
**Subsite**					
**Nakhon Phanom**	146 (65.5)	58 (59.2)	438 (66.5)	0.83	0.17
**Sa Kaeo**	77 (34.5)	40 (40.8)	221 (33.5)		
**Age**					
**28 days–5 months**	37 (16.6)	18 (18.4)	91 (13.8)	0.77	0.58
**6–11 months**	50 (22.4)	19 (19.4)	153 (23.2)		
**12–23 months**	69 (30.9)	30 (30.6)	217 (32.9)		
**24–59 months**	67 (30.0)	31 (31.6)	198 (30.0)		
**Sex**					
**Male**	134 (60.1)	55 (56.1)	336 (51.0)	**0.02**	0.34
**Female**	89 (39.9)	43 (43.9)	323 (49.0)		
**Season of enrollment**					
**Rainy (June–October**)	132 (59.2)	68 (69.4)	293 (44.5)	**<0.001**	**<0.001**
**Mild (November–February**)	55 (24.7)	21 (21.4)	214 (32.5)		
**Dry (March–May**)	36 (16.1)	9 (9.2)	152 (23.1)		
**Mother’s education**					
**No formal education/did not complete primary school**	12 (5.4)	5 (5.1)	28 (4.3)	0.91	0.42
**Completed primary school**	61 (27.5)	20 (20.4)	187 (28.8)		
**Completed lower secondary school**	67 (30.2)	31 (31.6)	200 (30.8)		
**Completed higher secondary school and above**	82 (36.9)	42 (42.9)	235 (36.2)		
**Monthly income (baht**)					
**0–2000 (0–65 USD**^c^)	21 (9.9)	5 (5.2)	30 (4.6)	**0.003**	0.29
**2001–7000 (65–229 USD**^c^)	77 (36.3)	37 (38.5)	199 (30.7)		
**>7000 (>229 USD**^c^)	114 (53.8)	54 (56.3)	419 (64.7)		
**Median (IQR) # people living in same household**	5.0 (4.0, 6.0)	5.0 (4.0, 6.0)	5.0 (4.0, 6.0)	0.32	0.96
**Median (IQR) # people sleeping in same room as child**	3.0 (3.0, 4.0)	3.0 (3.0, 4.0)	3.0 (3.0, 3.0)	**0.003**	0.10
**Median (IQR) # children <10 years in same household**	2.0 (1.0, 2.0)	2.0 (1.0, 2.0)	2.0 (1.0, 2.0)	0.11	0.07
**Number of DTP doses received**					
**None**	15 (6.9)	10 (10.4)	17 (2.6)	0.05	**0.007**
**1**	16 (7.4)	5 (5.2)	40 (6.1)		
**2**	29 (13.4)	14 (14.6)	63 (9.6)		
**3+**	157 (72.4)	67 (69.8)	535 (81.7)		
**DTP fully vaccinated for age** ^d^					
**<1 year old**	59 (70.2)	23 (62.2)	215 (88.5)	**0.001**	**0.002**
**≥1 year old**	122 (91.7)	54 (91.5)	405 (98.3)	**0.001**	**0.007**
**Received 1+ measles doses (children >10 months only**)	134 (93.7)	59 (93.7)	430 (97.3)	0.10	0.22
**Influenza vaccination (current season**)^e^	1 (0.5)	0 (0)	2 (0.5)	0.99	0.92
**Malnutrition (WHO weight-for-age Z scores**)					
**≥ −2 Z scores**	163 (73.1)	64 (65.3)	613 (93.0)	**<0.001**	**<0.001**
**−3 ≤ Z scores < −2**	28 (12.6)	16 (16.3)	41 (6.2)		
**< −3 Z scores**	32 (14.4)	18 (18.4)	5 (0.8)		
**Prior antibiotic use**					
**Serum antibiotic activity**	42 (19.0)	19 (19.4)	5 (0.9)	**<0.001**	**<0.001**
**Administered at study hospital before blood collection**^f^	28 (12.6)	14 (14.3)	n/a	—	—
**Any documented antibiotic pretreatment before blood collection**^g^	68 (30.5)	30 (30.6)	10 (1.6)	**<0.001**	**<0.001**
**Parental report of antibiotics**	104 (47.1)	53 (54.6)	55 (8.4)	**<0.001**	**<0.001**
**Any of above**	131 (58.7)	65 (66.3)	57 (9.7)	**<0.001**	**<0.001**
**CXR available**	203 (91)	98 (100.0)	n/a	—	—
**CXR result**					
**Consolidation only**	28 (13.8)	28 (28.6)	n/a	—	—
**Other infiltrate only**	56 (27.6)	56 (57.1)	n/a	—	—
**Both consolidation and other infiltrate**	14 (6.9)	14 (14.3)	n/a		
**Normal**	96 (47.3)	0 (0)	n/a	—	—
**Uninterpretable**	9 (4.4)	0 (0)	n/a	—	—
**Respiratory tract illness**^h^ (controls only)	n/a	n/a	254 (38.5)	—	—
**Duration of illness** ^i^					
**0–2 days**	99 (44.4)	38 (38.8)	n/a	—	—
**3–5 days**	98 (43.9)	48 (49.0)	n/a	—	—
**>5 days**	26 (11.7)	12 (12.2)	n/a	—	—
**Oxygen use at admission**	64 (28.7)	36 (36.7)	n/a	—	—
**Hypoxemia** ^j^	53 (23.8)	28 (28.6)	n/a	—	—
**Tachypnea** ^k^	168 (78.1)	74 (80.4)	48 (7.3)	**<0.001**	**<0.001**
**Tachycardia** ^l^	100 (44.8)	47 (48.0)	n/a	—	—
**Very severe pneumonia**	51 (22.9)	20 (20.4)	n/a	—	—
**Head nodding**	8 (3.6)	3 (3.1)	n/a	—	—
**Central cyanosis**	7 (3.1)	6 (6.1)	n/a	—	—
**Multiple (>1) or prolonged (≥15 minutes) convulsions**	23 (10.3)	4 (4.1)	n/a	—	—
**Lethargy**^m^	9 (4.0)	5 (5.1)	n/a	—	—
**Unable to feed**	8 (3.6)	4 (4.1)	n/a	—	—
**Vomiting everything**	8 (3.6)	3 (3.1)	n/a	—	—
**Crackles**	138 (61.9)	67 (68.4)	n/a	—	—
**Audible wheeze**	9 (4.0)	2 (2.0)	n/a	—	—
**Wheeze on auscultation**	102 (45.7)	38 (38.8)	n/a	—	—
**Grunting**	6 (2.7)	4 (4.1)	n/a	—	—
**Nasal flaring**	115 (51.6)	46 (46.9)	n/a	—	—
**Elevated temperature (≥38°C**)	122 (54.7)	55 (56.1)	n/a	—	—
**Leukocytosis** ^n^	119 (53.6)	48 (49.5)	183 (30.5)	**<0.001**	**<0.001**
**CRP ≥40 mg/L**	36 (17.7)	22 (24.2)	n/a	—	
**Prematurity (gestational age <37 weeks**)	45 (20.6)	25 (26.3)	51 (8.1)	**0.01**	**<0.001**
**Comorbidities, any**	54 (24.2)	33 (33.7)	n/a	—	—
**Heart disease (congenital or acquired**)^o^	20 (9.0)	15 (15.3)	n/a	—	—
**Developmental delays/congenital abnormality**	19 (8.5)	12 (12.2)	n/a	—	—
**Severe malnutrition**^p^	25 (11.2)	12 (12.2)	n/a	—	—
**Thalassemia**	6 (2.7)	3 (3.1)	n/a	—	—
**Other**^q^	2 (0.9)	2 (2.0)	n/a	—	—
**Multiple comorbidities**	17 (7.6)	11 (11.2)	n/a	—	—
**Previous pneumonia admission**	79 (35.7)	38 (38.8)	36 (5.5)	**<0.001**	**<0.001**
**Previous diagnosis of wheeze or asthma**	45 (20.3)	21 (21.4)	40 (6.1)	**<0.001**	**<0.001**
**Evidence of persistent severity** ^r^					
**Any evidence**	45 (20.2)	24 (24.5)	n/a	—	—
**Danger signs at 48 hours**	5 (2.5)	3 (3.2)	n/a	—	—
**Change in antibiotics at 48 hours**^s^	23 (10.3)	11 (11.2)	n/a	—	—
**Oxygen saturation <90% at 48 hours**	3 (1.3)	2 (2.0)			
**Mechanical ventilation at 48 hours**	19 (8.5)	12 (12.1)	n/a	—	—
**Died in hospital or within 30 days of discharge**	9 (4.1)	5 (5.2)	n/a	—	—
**Died in hospital**	3 (1.3)	2 (2.0)	n/a	—	—
**Died within 30 days of discharge**	6 (2.8)	3 (3.1)	n/a	—	—

^a^CXR+ cases: finding of consolidation, other infiltrate or both on chest radiograph.

^b^*P* values for categorical variables are type III *P* values calculated using logistic regression adjusting for age.

^c^US dollar equivalents based on exchanges rates from January 1, 2013, the midpoint of the PERCH Study in Thailand.

^d^For children <1 year, DTP fully vaccinated defined as: received at least 1 dose and up-to-date for age based on the child’s age at enrollment, doses received, and country schedule (allowing 4-week window each for dose). For children >1 year, defined as 3+ doses.

^e^Receipt of influenza vaccine for current or most recent influenza season. Denominator restricted to children eligible to receive free flu vaccination per Thailand Ministry of Public Health recommendations, that is, children with a comorbidity or previous diagnosis of asthma or wheeze, and children of 6–23 months of age. Children who were 6–23 months old at enrollment or at any time during the past 12 months were considered eligible. Influenza season is June–August in Thailand with influenza vaccination available starting in June (all cases, N = 182; CXR+ cases, N = 84; controls, N = 442).

^f^Reported by clinician.

^g^Presence of antibiotics by serum, antibiotics at the referral hospital, clinician report of antibiotics before blood collection or antibiotics before blood collection based on time of blood collection and time of antibiotic administration. Only criterion applicable to controls is serum.

^h^Respiratory tract illness defined as (1) cough and/or runny nose or (2) at least 1 of ear discharge, wheezing or difficulty breathing in the presence of either a temperature of ≥38°C within past 48 hours or a history of sore throat.

^i^Duration of illness defined as number of days of cough, fever, difficulty breathing or wheeze, whichever is longest prior to date of admission.

^j^Oxygen saturation <92% on room air at admission or oxygen requirement (if no room air reading available).

^k^Tachypnea: RR ≥ 60 for age <2; RR ≥ 50 for age 2–11 months; RR ≥ 40 for age 12+.

^l^Tachycardia: 0–11 months > 160 beats per minute (BPM); 12–35 months > 150 BPM; 36–59 months > 140 BPM.

^m^Lethargy: responds to voice, pain or unresponsive (V, P or U on AVPU scale).

^n^Leukocytosis defined as white blood cell count ≥15,000 per µL

^o^Heart disease includes any of the following noted on admission or discharge: cardiac lesions, congenital cardiac lesions, congenital heart disease, patent ductus arteriosus, valvular heart defect, atrial septal defect, cardiomegaly.

^p^WHO weight-for-height Z score < −3 SDs, or midupper arm circumference <115 mm, or presence of pedal edema on clinical assessment.

^q^Other comorbidities include Lennox–Gastault syndrome and megalocephaly.

^r^Evidence of persistent severity defined as died in hospital or within 30 days of discharge, discharged moribund, mechanical ventilation at 48 hours, change in antibiotics at 48 hours, oxygen saturation <90% at 48 hours, or presence of a danger sign (head nodding, central cyanosis, multiple or prolonged convulsions, lethargy, inability to feed or vomiting everything) at 48 hours.

^s^Change in antibiotics between 24- and 48-hour follow up due to any of the following: not responding to initial therapy, new finding on CXR, new diagnostic test result or allergic reaction to medication.

IQR indicates interquartile range; PERCH, Pneumonia Etiology Research for Child Health; DTP, diphtheria–pertussis–tetanus vaccine; WHO, World Health Organization; CXR, chest Radiograph; CRP, c-reactive protein.

Bold indicates significance of *P* < 0.05.

Thirty percent of cases had evidence of antibiotic treatment before blood collection compared with <2% of controls (*P* < 0.001). Symptoms of respiratory tract infections (RTIs) were common among controls (38.5%), but only 1.5% (*n* = 10) met the WHO definition for nonsevere pneumonia (ie, cough or difficulty breathing and tachypnea). Many (45.0%) of the RTIs among controls were defined by runny nose only.

Danger signs, the defining criteria for very severe pneumonia, were present in 22.9% of cases (20.4% among CXR+ cases) with convulsions being most common (10.3% of all cases). Of very severe cases, many (33.3%) did not have a CXR available; of those with a CXR, 35.3% (12/34) had normal findings.

CXR+ cases were not easily identifiable by other clinical characteristics. Hypoxemia, fever and tachypnea were only slightly more common among CXR+ cases than among cases overall (Table 1). Prior hospital admission for pneumonia (35.7%) and previous asthma diagnosis (20.3%) were common among cases. Comorbidities were documented in 24.2% of cases (33.7% among CXR+ cases). Over 20% of cases were born before <37 weeks gestation, exceeding the 8.1% of controls born prematurely.

Approximately 20% of cases had evidence of persistent severity indicated by the need for antibiotic change, mechanical ventilation, oxygen saturation <90% or the presence of danger signs 48 hours postadmission or by death (Table 1). Of 220 cases with known vital status by the 30-day follow-up, 9 (4.1%) died (3 died in hospital and 6 died within 30 days after discharge). Six of these 9 children had no danger signs at admission, 7 had at least 1 comorbidity (5 had developmental delay, 3 heart disease, 2 severe malnutrition, and 1 thalassemia), 5 were CXR+ while 4 had a normal CXR and 5 were <6 months old (Table, Supplemental Digital Content 1, http://links.lww.com/INF/E23).

### Pathogen Detection and Etiology Results

Five (2.2%) of the 223 HIV-uninfected cases had positive blood cultures (2 of 98 CXR+), 2 of which (*M. catarrhalis* and *S. pyogenes*) were also NP/OP PCR positive for influenza B (Table 2). Bacteremic cases accounted for 2 (22%) of the 9 deaths. The one HIV+ case, which was not included in other analyses, had nontyphoidal *Salmonella* serogroup D isolated by blood culture. Three cases (1.3%; all CXR-normal) and 5 controls (0.8%) had *S. pneumoniae* detected by PCR in whole blood specimens (OR 1.77; 95% CI: 0.42, 7.50); none of the cases were CXR+.

**TABLE 2.** Cases With a Pathogen Detected by Blood Culture—PERCH, Thailand, 2012–2013^a^

Age (months)	Pneumonia Severity	Pathogen Detected By Blood Culture	Pathogens Detected By NP/OP PCR	CXR Result	Antibiotic Pretreatment Before Specimen Collection^b^	Comorbidities/Prematurity	Died in Hospital or Within 30 Days of Discharge	Duration of Hospitalization (days)
2	Severe	*Escherichia coli*	CMV*Haemophilus influenzaePneumocystis jirovecii*	Not performed	No	Premature (36 weeks)	No	7
2	Severe	*Pseudomonas aeruginosa*	*Moraxella catarrhalis*, *Staphylococcus aureus*	Normal	Yes	No	Yes (in hospital)	3
13	Severe	*M. catarrhalis*	CMV, *H. influenzaeM. catarrhalisStreptococcus pneumoniae*RSV	Other infiltrate	Yes	Congenital heart disease, malnutrition	No	6
39	Very severe	*M. catarrhalis*	CMVinfluenza B*M. catarrhalisS. pneumoniae*	Normal	No	No	No	7
45	Severe	*Streptococcus pyogenes*	CMVinfluenza B	Consolidation	Yes	Down syndrome, cerebral palsy, congenital hypothyroidism	Yes (in hospital)	5

^a^The one HIV+ case in Thailand had nontyphoidal Salmonella detected on blood culture and is not included in the table. This case had very severe pneumonia, both consolidation and other infiltrate detected on chest radiograph, CMV, *H. influenzae* and *P. jirovecii* detected by NPPCR, and was discharged after 29 days in hospital.

^b^Presence of antibiotics by serum, antibiotics at the referral hospital, clinician report of antibiotics before specimen collection or antibiotics before specimen collection based on time. Of specimen collection and time of antibiotic administration.

NP/OP indicates nasopharyngeal/oropharyngeal; PCR, polymerase chain reaction; CMV, cytomegalovirus; RSV, respiratory syncytial virus; HIV, human immunodeficiency virus.

Two cases, from Sa Kaeo province, had MTB confirmed by induced sputum culture: one was a 3-year-old with no underlying conditions and cough for 5 days; the other was a 4-year-old with Down syndrome, atrial septal defect, congenital hypothyroidism (on therapy) and cough for 3 days. Both had crepitation on chest auscultation, no danger signs, CXR+ and positive NP/OP specimens for another pathogen (*Mycoplasma pneumoniae*, RSV) uncommonly (≤3%) found in controls. Each child had a grandparent with presumptive pulmonary tuberculosis.

Over 96% of both cases and controls had at least 1 pathogen detected by NP/OP PCR (Table 3), and multiple detections were common with 2 or more pathogens detected in 86% of cases and 88% of controls (Table, Supplemental Digital Content 2, http://links.lww.com/INF/E24). None of the bacterial pathogens were significantly positively (ie, OR > 1) associated with case status, while 6 viral pathogens were: RSV, parainfluenza 1, influenza B, human metapneumovirus (HMPV) A/B, coronavirus OC43 and rhinovirus (Table 3). RSV was the only pathogen with both a high prevalence in cases (22.9%) and a strong association (OR 13.9; 95% CI: 7.7, 25.2). Parainfluenza 1 (2.2%; OR 12.5) and influenza B (1.3%; OR 9.5) had strong associations but low prevalence, rhinovirus had the reverse (19.3%; OR 2.3) and coronavirus OC43 (3.1%; OR 2.7) and HMPV A/B (3.6%; OR 3.7) were low for both. When restricted to the 98 CXR+ cases, prevalence and association with case status remained generally similar for all pathogens, but only RSV and HMPV A/B remained statistically significant due to the smaller sample size.

**TABLE 3.** Pathogen Detection in Nasopharyngeal/Oropharyngeal Specimens Among Cases and Controls—PERCH, Thailand, 2012–2013

	All Cases (N = 223)	CXR+ Cases (N = 98)	Controls (N = 657)	Adjusted OR (95% CI)^a^	
				All Cases vs. Controls	CXR+ Cases vs. Controls
**Any pathogen**	215 (96.4)	97 (99.0)	634 (96.5)	0.90 (0.34, 2.43)	2.97 (0.38, 23.56)
**Median (IQR) number of organisms**	3.0 (2.0, 4.0)	3.0 (2.0, 4.0)	3.0 (2.0, 4.0)		
**Bacteria**					
Any bacteria	191 (85.7)	86 (87.8)	587 (89.3)		
*Streptococcus pneumonia*					
Any positivity	121 (54.3)	59 (60.2)	406 (61.8)	0.74 (0.51, 1.07)	0.95 (0.56, 1.63)
≥6.9 log_10_ copies/mL	3 (1.3)	2 (2.0)	8 (1.2)	0.71 (0.13, 3.84)	1.19 (0.16, 9.01)
PCV13-type^b^	3 (1.3)	2 (2.0)	6 (0.9)	1.15 (0.14, 9.30)	2.60 (0.22, 31.20)
Non PCV13-type^b^	1 (0.4)	1 (1.0)	3 (0.5)	0.36 (0.01, 9.36)	0.22 (0.01, 9.38)
*Haemophilus influenza*					
Non-b	74 (33.2)	29 (29.6)	262 (39.9)	0.83 (0.58, 1.20)	0.65 (0.38, 1.13)
Non-b ≥ 5.9 log_10_ copies/mL	20 (9.0)	7 (7.1)	70 (10.7)	1.21 (0.69, 2.13)	1.01 (0.43, 2.41)
Type b	5 (2.2)	3 (3.1)	15 (2.3)	0.80 (0.23, 2.73)	1.57 (0.35, 7.01)
Type b ≥ 5.9 log_10_ copies/mL	0 (0)	0 (0)	0 (0)	—	—
*Staphylococcus aureus*	27 (12.1)	14 (14.3)	107 (16.3)	0.69 (0.41, 1.15)	0.81 (0.41, 1.62)
*Chlamydia pneumoniae*	0 (0)	0 (0)	1 (0.2)	0.00 (0.00, >999)	0.00 (0.00, >999)
*Moraxella catarrhalis*	101 (45.3)	44 (44.9)	362 (55.1)	0.71 (0.50, 1.01)	0.72 (0.44, 1.19)
*Mycoplasma pneumoniae*	10 (4.5)	6 (6.1)	20 (3.0)	1.23 (0.51, 2.98)	1.90 (0.64, 5.66)
Salmonella species	0 (0)	0 (0)	2 (0.3)	0.00 (0.00, >999)	0.00 (0.00, >999)
Legionella	0 (0)	0 (0)	0 (0)	—	—
*Bordetella pertussis*	0 (0)	0 (0)	3 (0.5)	0.00 (0.00, >999)	0.00 (0.00, >999)
**Fungi**					
*Pneumocystis jirovecii*	5 (2.2)	3 (3.1)	32 (4.9)	0.52 (0.19, 1.44)	0.73 (0.20, 2.66)
*P. jirovecii* ≥4 log_10_ copies/mL	0 (0)	0 (0)	7 (1.1)	0.00 (0.00, >999)^c^	0.00 (0.00, >999)^c^
**Viruses**					
Any virus	190 (85.2)	88 (89.8)	540 (82.2)		
Adenovirus	31 (13.9)	13 (13.3)	140 (21.3)	0.72 (0.45, 1.15)	0.68 (0.34, 1.34)
CMV	103 (46.2)	41 (41.8)	402 (61.2)	**0.57 (0.39, 0.83**)	**0.54 (0.32, 0.92**)
CMV ≥4.9 log_10_ copies/mL	30 (13.5)	10 (10.2)	110 (16.7)	0.64 (0.38, 1.07)	**0.35 (0.16, 0.79**)
Coronavirus 43	7 (3.1)	3 (3.1)	13 (2.0)	**2.71 (1.01, 7.26**)	2.26 (0.57, 9.02)
Coronavirus 63	3 (1.3)	1 (1.0)	12 (1.8)	1.25 (0.33, 4.64)	0.92 (0.11, 7.53)
Coronavirus HKU	0 (0)	0 (0)	2 (0.3)	0.00 (0.00, >999)	0.00 (0.00, >999)
Coronavirus 229	0 (0)	0 (0)	2 (0.3)	0.00 (0.00, >999)	0.00 (0.00, >999)
HBOV	40 (17.9)	17 (17.3)	123 (18.7)	1.07 (0.68, 1.68)	1.21 (0.64, 2.29)
HMPV A/B	8 (3.6)	4 (4.1)	10 (1.5)	**3.67 (1.35, 9.92**)	**4.08 (1.14, 14.56**)
Influenza A	4 (1.8)	1 (1.0)	0 (0)	>999 (0.00, >999)^d^	>999 (0.00, >999)^d^
Influenza B	3 (1.3)	1 (1.0)	2 (0.3)	**9.54 (1.51, 60.18**)	7.57 (0.63, 91.10)
Influenza C	0 (0)	0 (0)	0 (0)	—	—
Parainfluenza 1	5 (2.2)	1 (1.0)	2 (0.3)	**12.53 (2.28, 68.82**)	8.34 (0.70, 98.93)
Parainfluenza 2	0 (0)	0 (0)	1 (0.2)	0.00 (0.00, >999)	0.00 (0.00, >999)
Parainfluenza 3	6 (2.7)	3 (3.1)	15 (2.3)	1.94 (0.71, 5.28)	2.48 (0.67, 9.21)
Parainfluenza 4	2 (0.9)	1 (1.0)	9 (1.4)	1.10 (0.22, 5.42)	1.21 (0.15, 10.05)
PV/EV	21 (9.4)	7 (7.1)	57 (8.7)	1.47 (0.82, 2.64)	0.94 (0.39, 2.27)
Rhinovirus	43 (19.3)	15 (15.3)	93 (14.2)	**2.30 (1.48, 3.57**)	1.57 (0.82, 3.01)
RSV	51 (22.9)	30 (30.6)	19 (2.9)	**13.91 (7.66, 25.25**)	**20.48 (10.17, 41.26**)

^a^OR adjusted for age (months). ORs for individual pathogens also adjusted for all other pathogens.

^b^Pneumococcal serotypes were determined by PCR deduction followed by Quellung reaction if the results were mixed or ambiguous.

^c^Fisher exact test *P* value is 0.201 for all cases versus controls and 0.604 for CXR+ cases versus controls.

^d^Fisher exact test *P* value is 0.004 for all cases versus controls and 0.253 for CXR+ cases versus controls.

PERCH indicates Pneumonia Etiology Research for Child Health; OR, odds ratio; CXR+, chest Radiograph positive; CMV, cytomegalovirus; HBOV, human bocavirus; HMPV, human metapneumovirus; PV/EV, parechovirus/enterovirus; RSV, respiratory syncytial virus.

Bold indicates significance of *P* < 0.05.

The small sample size for CXR+ cases (n = 98), the small number of positive blood cultures among them (n = 2) and the large proportion of children pretreated with antibiotics limit the extent to which the etiology of the severe/very severe pneumonia cases can be estimated in Thailand. Integration of the results from the various tests using the PIA model suggests that a large etiologic fraction of CXR+ cases was attributed to RSV (34.6%; 95% CrI: 22.2, 49.8), followed by TB (10.4%; 95% CrI: 1.6, 25.6) (Figure, Supplemental Digital Content 3, http://links.lww.com/INF/E25 and Table, Supplemental Digital Content 4, http://links.lww.com/INF/E26), but most cases had no clear etiology (66% had less than a 50% probability attributed to a specific pathogen, data not shown). The etiologic fractions attributed to influenza and parainfluenza were both small in the years of this study (Influenza A, B or C: 1.5%; 95% CrI: 0.01, 5.6; Parainfluenza 1, 2, 3 or 4: 3.0%; 95% CrI: 0.08, 9.2). The cumulative estimate for etiology attributed to any virus had a wide credible interval (etiologic fraction 51%; 95% CrI: 34, 70), as did the cumulative estimate for any non-TB bacterial pathogen (30.9%; 95% CrI: 12.9, 50.8). The 10 focus pathogens among all PERCH sites (RSV, parainfluenza, HMPV, pneumococcus, rhinovirus, *Mycobacterium tuberculosis* (TB), *H. influenzae*, *Staphylococcus aureus*, influenza, *P. jirovecii*)^15^ accounted for 61.6% (95% CrI: 0, 80.5) of CXR+ cases [64.1% (95% CrI: 0, 78.5) of all cases] in Thailand (Table, Supplemental Digital Content 4, http://links.lww.com/INF/E26).

## DISCUSSION

After screening all children 1–59 months of age presenting at 2 provincial hospitals in Thailand over a 2-year period and enrolling >90% (n = 224) of eligible severe and very severe pneumonia cases, RSV accounted for the greatest fraction of disease followed by TB. We estimated 34.6% (95% CrI: 22.2, 49.8) of the etiologic contribution to pneumonia among the 98 CXR+ cases was due to RSV and 10.4% (95% CrI: 1.6, 25.6) was due to TB. The etiologic contribution of other pathogens at the Thailand site is less clear due to the small number of cases, and the large 95% credible intervals reflect the uncertainty in the results.

RSV NP/OP positivity in community controls was rare, lending credibility that RSV detection in cases provided strong evidence for RSV-associated pneumonia. In a previous evaluation of hospitalized ALRI cases in these same provinces, 20% had RSV detected in the NP (25% of CXR+ cases),^11^ similar to the 23% in PERCH severe and very severe pneumonia cases (31% in CXR+ cases). RSV remains a leading cause of ALRI hospitalizations in Thailand, including clinically severe cases. The dominance of RSV was consistent across all PERCH sites in both Africa and Asia, providing compelling evidence for the importance of RSV across regions with varying access to care, overall disease burden and socioeconomic status.^15^

We estimated that TB accounted for 10% (95% CI: 1.6, 26) of CXR+ severe and very severe pneumonia cases among children <5 years old in Thailand. This is higher than the observed 2% (n = 2) that tested positive by induced sputum culture, because the integrated analysis accounts for imperfect sensitivity of TB testing, by assuming 3.3–10 additional TB cases for every case of TB detected. The etiologic fraction of cases attributed to TB in other PERCH sites ranged from 1.8% (Kenya) to 14% (South Africa).^15^ In routine care conditions, it is very unlikely that the 2 TB-positive cases would have been identified because the systematic collection of induced sputum and TB culture are not routinely done. Underdiagnosis of TB among adults has been demonstrated previously in this population,^42^ and given the substantial difficulties with TB diagnosis among young children,^43^ we presume even more so among children. In the cases with TB isolated, we cannot confirm whether TB was the cause of the acute pneumonia event, or an underlying infection predisposing to disease caused by another pathogen, since the cases were also NP/OP-positive for *M. pneumoniae* or RSV. Regardless of the role of TB in acute pneumonia, our findings support evaluation for TB among children hospitalized with respiratory illness in high-burden TB settings like Thailand.^44,45^

Rhinovirus had a low (4.5%; 95% CrI: <0.01–15.7) etiologic fraction estimate among CXR+ cases, but it was higher (16%; 95% CrI: 7.7–26) among all PERCH cases in these Thai sites, though credible intervals are overlapping. Detection of rhinovirus in the NP/OP in children <5 years in PERCH (all cases: 19%; CXR+ cases: 15%) was similar to that in our prior work in this population where rhinovirus was detected in 18%–29% of children hospitalized with ALRI but also commonly detected among controls (12%–14%).^10,11^ Findings from PERCH and from previous studies indicate that rhinovirus may have a role in hospitalized respiratory illness in young Thai children but a less prominent role in radiographically confirmed pneumonia.^11^ The PERCH case–control study design limits our ability to elucidate fully the potential causal role for rhinovirus.

The NP/OP prevalence of parainfluenza and HMPV among cases in Thailand was lower than in most other PERCH sites (lower than all other sites for CXR+ cases). Previous work in this population found a slightly higher prevalence of parainfluenza virus among ALRI cases <5 years of age than we found in PERCH (serotypes 1–3: 9.1% vs. 5%) and similar prevalence of HMPV. Lower prevalence of both pathogens among outpatient controls in the previous work resulted in higher ORs compared with PERCH results.^11,46^ The higher parainfluenza and HMPV prevalence among PERCH controls may be attributable to increased sensitivity of newer PCR testing and the choice of control group. Influenza was uncommonly detected at any of the PERCH sites, including Thailand, but this contrasts with previous work in Thailand that identified influenza as a leading pathogen among ALRI cases.^46^ It is important to note that our previous work was not limited to severe pneumonia or CXR+ pneumonia. Further, parainfluenza activity is cyclical,^47^ and influenza activity varies substantially from year to year^8^; influenza surveillance revealed relatively low activity from 2011 through 2013 in Thailand.^48,50^

Given there were only 98 CXR+ severe or very severe pneumonia cases in the 2-year study period, precise estimation of the etiologic fraction for individual bacteria was not possible. The observed 2%–3% blood culture positivity was consistent with previously reported positivity of 1.8% (145/7975) among children <5-years-old hospitalized with ALRI.^11^ Among those ALRI cases, *S. pneumoniae*, nontyphoid *Salmonella* species, *H. influenzae*, *Escherichia coli*, *Acinetobacter baumannii* and *S. aureus* were the most common bacterial bloodstream infections.^11^ However, positive blood cultures in the current study identified pathogens less commonly associated with childhood pneumonia, but inference from this finding is limited by small numbers; 3 of the 5 positive cultures occurred in children with comorbidities or prematurity. *M. catarrhalis* bacteremia, identified in 2 cases who were also positive for RSV or influenza B, has been described, albeit infrequently, in children with acute respiratory illness, including pneumonia.^52,53^

It is notable that no PERCH case had *S. pneumoniae* detected by blood culture despite the near lack of PCV use, and only 3 (0 CXR+) cases were blood positive by PCR. However, there is evidence that *S. pneumoniae* caused disease in children <5 years in these same provinces before, during and after the PERCH study period. Before PERCH, we observed an annual incidence of *S. pneumoniae* bacteremia hospitalizations of 11.1 per 100,000 persons (95% CI: 7.9, 15.1), which was likely substantially underestimated due to preculture antibiotic use.^52,53^ During PERCH (2012–2013), concurrent bloodstream infection surveillance in these provinces identified 6 cases of *S. pneumoniae* bacteremia in children <5 years of age, 3 of whom were screened for PERCH but did not meet the severe or very severe pneumonia criteria and 3 of whom were treated at hospitals not participating in PERCH. After PERCH ended, 10 children <5-years-old from Nakhon Phanom and Sa Kaeo provinces (8 at PERCH study hospitals) had *S. pneumoniae* bacteremia in 2014 and 9 in 2015, which translates to incidence rates of 11.6 and 10.5 per 100,000 for 2014 and 2015, respectively (unpublished data). Among persons ≥5 years, 121 pneumococcal bacteremia cases occurred in those 2 years in the study provinces. Similarly, there were 9 *H. influenzae* bacteremia cases among children <5-years-old in these provinces during the PERCH study period, but 5 were treated at other hospitals and the remaining 4 *H. influenzae* cases did not meet the enrollment criteria. This provides additional evidence that both *S. pneumoniae* and *H. influenzae* play important roles in pneumonia and severe disease among young children in Thailand. The lack of culture-confirmed pneumococcus among severe and very severe pneumonia cases in PERCH in Thailand is possibly due to good access to care and early antibiotic administration, which may help prevent more severe forms of pneumococcal disease. In the PERCH Study overall, antibiotic administration before blood culture collection was associated with a 45% reduction in culture yield.^38^ Additionally, pneumococcal bacteremia is a relatively uncommon event in general, and given the insensitivity of blood culture, it would not be unusual to have periods with no positive cultures.

It is also notable that approximately one-quarter of all Thailand cases (34% of CXR+ cases) had comorbidities, which is comparable with the 11%–34% with comorbidities at the other PERCH sites. Comorbidities were also an important contributing factor to fatalities in Thailand [78% (7/9) of fatal cases had a comorbidity; developmental delay (5/7) in particular], as was observed in other PERCH sites (range 44% in The Gambia and Zambia to 67%–80% in Mali, Kenya and Bangladesh).

### Study Limitations

The PERCH Study case–control design and the collection of specimens at admission only after the onset of infection does not allow us to delineate the order of organism acquisition or differentiate between nonpathogenic organisms that were present before symptom onset and organisms that were part of the causal chain for severe pneumonia hospitalization. Furthermore, the current PIA model estimates the contribution of single pathogens but not copathogen events. For cases with multiple organisms detected, the etiology for an individual case is distributed across the multiple organisms according to the strength of the evidence for each organism (ie, its prevalence among cases and its NP/OP PCR OR), a priori assumptions regarding sensitivity and specificity, and whether it was detected in one versus multiple specimens.^39^ The etiologic contribution of some pathogens may be underestimated if they contributed to a coinfection, because the sum of the pathogen probabilities for an individual case must be 100%. In addition, although we enrolled cases over a 24-month period, there were an insufficient number of cases to evaluate seasonality for most pathogens so there is potential to under- or overestimate the importance of pathogens with periodic epidemics. Further, the 2 years of study enrollment may not have been representative of other years; in fact, Thailand, like many sites, observed fewer influenza cases than in nonstudy years. These issues apply to all PERCH Study sites,^15^ but lower-than-expected enrollment in Thailand (224 enrolled vs. 500 expected) limited our ability to estimate precisely pathogen-specific etiologic fractions, especially when restricted to CXR+ or stratified by severity or age. Our expected sample size was largely based on observed numbers of hospitalized ALRI cases who received supplemental oxygen at these hospitals during prior years of pneumonia surveillance.^7^

The previously observed high incidence of hospitalized ALRI (5772 per 100,000 child-years [95% CI: 5707, 5837]),^11^ together with the relative infrequency of severe and very severe pneumonia and low case fatality, suggest that early access to care, including antibiotics, hospitalization and supplemental oxygen use likely results in a low proportion of children progressing to severe or very severe pneumonia. Previous healthcare utilization surveys in these provinces confirmed that the majority of children <5 years of age with ALRI were taken to hospital^7,54^ and from 2006 to 2010, UNICEF estimated that 84% of Thai children <5 years of age with suspected pneumonia were taken to a health care provider.^55^

## CONCLUSIONS

In Thailand, RSV was the predominant pneumonia pathogen, as it was at all sites, accounting for approximately one-third of cases and confirming the urgent need to develop and evaluate new prevention strategies that are feasible to implement globally. The highly standardized PERCH study methods give us confidence that etiology differences are not the result of differences in study implementation practices but could be related to differences in access to care and early interventions, pathogen ecology or possibly biologic differences that increase or decrease susceptibility to certain pathogens. Despite differences, important similarities in etiology findings between Thailand and other PERCH sites suggest that global control strategies, including case management algorithms and immunization priorities, based on overall PERCH findings are relevant to Thailand and similar settings.

## ACKNOWLEDGMENTS

We would like to thank Somyos Sricharanai, Peera Aareerat, Sirirat Makprasert, Possawat Jorakate, Anchalee Jatapai, Patranuch Sapchookul, Prasong Srisaengchai and Sununta Henchaichon for their contribution to this project. We offer sincere thanks to the study participants and their families. We recognize the efforts of the following groups during the study development, conduct and analysis phases: Pneumonia Methods Working Group, PERCH Expert Group, PERCH contributors, and the PERCH Chest Radiograph Reading Panel. We also acknowledge the substantial contributions of all members of the PERCH Study Group (see below).

Johns Hopkins Bloomberg School of Public Health, Baltimore, Maryland: Orin S. Levine (Former PI, current affiliation Bill & Melinda Gates Foundation, Seattle, Washington), Andrea N. DeLuca, Amanda J. Driscoll, Nicholas Fancourt, Wei Fu, E. Wangeci Kagucia, Ruth A. Karron, Mengying Li, Daniel E. Park, Qiyuan Shi, Zhenke Wu, Scott L. Zeger; The Emmes Corporation, Rockville, Maryland: Nora L. Watson; Nuffield Department of Clinical Medicine, University of Oxford, United Kingdom: Jane Crawley; Medical Research Council, Basse, The Gambia: Stephen R. C. Howie (site PI); KEMRI-Wellcome Trust Research Programme, Kilifi, Kenya: J. Anthony G. Scott (site PI and PERCH co-PI, joint affiliation with London School of Hygiene and Tropical Medicine, London, United Kingdom); Division of Infectious Disease and Tropical Pediatrics, Department of Pediatrics, Center for Vaccine Development, University of Maryland School of Medicine, Baltimore, Maryland and Centre pour le Développement des Vaccins (CVD-Mali), Bamako, Mali: Karen L. Kotloff (site PI); Medical Research Council: Respiratory and Meningeal Pathogens Research Unit and Department of Science and Technology/National Research Foundation: Vaccine Preventable Diseases, University of the Witwatersrand, Johannesburg, South Africa: Shabir A. Madhi (site PI); International Centre for Diarrhoeal Disease Research, Bangladesh (icddr,b): W. Abdullah Brooks (site PI); Boston University School of Public Health, Boston, Massachusetts and University Teaching Hospital, Lusaka, Zambia: Donald M. Thea (site PI); Canterbury Health Laboratories, Christchurch, New Zealand: Trevor P. Anderson, Joanne Mitchell, Shalika Jayawardena, Rose Watt.

## Supplementary Material



## References

[1] Adegbola RA. Childhood pneumonia as a global health priority and the strategic interest of the Bill & Melinda Gates Foundation. Clin Infect Dis. 2012;54(Suppl 2):S89–S92. 22403237 10.1093/cid/cir1051

[2] World Health Organization. WHO Media centre, Fact sheet, Children: Reducing Mortality. 2017. Available at: http://www.who.int/mediacentre/factsheets/fs178/en/. Accessed November 28, 2017.

[3] Liu L Oza S Hogan D. Global, regional, and national causes of under-5 mortality in 2000-15: an updated systematic analysis with implications for the Sustainable Development Goals. Lancet. 2016;388:3027–3035. 27839855 10.1016/S0140-6736(16)31593-8 PMC5161777

[4] Institute for Health Metrics and Evaluation (IHME). Global Burden of Disease Compare Data Visualization. Available at: http://vizhub.healthdata.org/gbd-compare. Accessed December 6, 2017.

[5] Scott JA Brooks WA Peiris JS. Pneumonia research to reduce childhood mortality in the developing world. J Clin Invest. 2008;118:1291–1300. 18382741 10.1172/JCI33947 PMC2276784

[6] Shann F. The management of pneumonia in children in developing countries. Clin Infect Dis. 1995;21(Suppl 3):S218–S225. 8749670 10.1093/clind/21.supplement_3.s218

[7] Jordan HT Prapasiri P Areerat P. A comparison of population-based pneumonia surveillance and health-seeking behavior in two provinces in rural Thailand. Int J Infect Dis. 2009;13:355–361. 18977679 10.1016/j.ijid.2008.07.014

[8] Simmerman JM Chittaganpitch M Levy J. Incidence, seasonality and mortality associated with influenza pneumonia in Thailand: 2005-2008. PLoS One. 2009;4: e7776. 19936224 10.1371/journal.pone.0007776 PMC2777392

[9] Naorat S Chittaganpitch M Thamthitiwat S. Hospitalizations for acute lower respiratory tract infection due to respiratory syncytial virus in Thailand, 2008-2011. J Infect Dis. 2013; 208 Suppl 3: S238–S245. 24265483 10.1093/infdis/jit456

[10] Fry AM Lu X Olsen SJ. Human rhinovirus infections in rural Thailand: epidemiological evidence for rhinovirus as both pathogen and bystander. PLoS One. 2011;6:e17780. 21479259 10.1371/journal.pone.0017780 PMC3066183

[11] Hasan R Rhodes J Thamthitiwat S. Incidence and etiology of acute lower respiratory tract infections in hospitalized children younger than 5 years in rural Thailand. Pediatr Infect Dis J. 2014;33:e45–e52. 24030346 10.1097/INF.0000000000000062 PMC4667718

[12] Driscoll AJ Karron RA Morpeth SC. Standardization of laboratory methods for the PERCH Study. Clin Infect Dis. 2017;64(suppl 3):S245–S252. 28575358 10.1093/cid/cix081 PMC5447855

[13] Levine OS O’Brien KL Deloria-Knoll M. The Pneumonia Etiology Research for Child Health Project: a 21st century childhood pneumonia etiology study. Clin Infect Dis. 2012;54(Suppl 2):S93–101. 22403238 10.1093/cid/cir1052 PMC3297546

[14] Deloria-Knoll M Feikin DR Scott JA. Identification and selection of cases and controls in the Pneumonia Etiology Research for Child Health Project. Clin Infect Dis. 2012;54(Suppl 2):S117–S123. 22403225 10.1093/cid/cir1066 PMC3297551

[15] Pneumonia Etiology Research for Child Health Study G. Causes of severe pneumonia requiring hospital admission in children without HIV infection from Africa and Asia: the PERCH multi-country case-control study. Lancet. 2019;394:757–779. 31257127 10.1016/S0140-6736(19)30721-4 PMC6727070

[16] O’Brien KL Baggett HC Brooks WA. Introduction to the Epidemiologic Considerations, Analytic Methods, and Foundational Results From the Pneumonia Etiology Research for Child Health Study. Clin Infect Dis. 2017;64(suppl 3):S179–S184. 28575368 10.1093/cid/cix142 PMC5447854

[17] Data TWB. Gross National Income per capita. Available at: https://data.worldbank.org/indicator/NY.GNP.PCAP.CN?end=2016&locations=TH. Accessed November 17, 2017.

[18] Council NEaSD. Gross Regional and Provincial Product (GPP). Available at: https://www.nesdc.go.th/nesdb_en/main.php?filename=national_account. Accessed December 18, 2017.

[19] Data TWB. Mortality rate, under-5 (per 1,000 live births). Available at: https://data.worldbank.org/indicator/SH.DYN.MORT?end=2012&locations=BD-GM-KE-ML-ZA-TH-ZM&start=2012&view=bar. Accessed November 17, 2017.

[20] UNAIDS. Country factsheets Thailand/2012. Available at: http://aidsinfo.unaids.org. Accessed November 17, 2017.

[21] Lolekha R Boonsuk S Plipat T. Elimination of Mother-to-Child Transmission of HIV-Thailand. MMWR Morb Mortal Wkly Rep. 2016;65:562–566. 27281244 10.15585/mmwr.mm6522a2

[22] Office HISR. Thailand’s Universal Coverage Scheme: Achievements and Challenges. Available at: http://www.hisro.or.th/main/download/10UCS_Eng.pdf. Accessed December 5, 2017.

[23] Owusu JT Prapasiri P Ditsungnoen D. Seasonal influenza vaccine coverage among high-risk populations in Thailand, 2010-2012. Vaccine. 2015;33:742–747. 25454853 10.1016/j.vaccine.2014.10.029 PMC4610807

[24] Kittikraisak W Suntarattiwong P Levy J. Influenza vaccination coverage and effectiveness in young children in Thailand, 2011-2013. Influenza Other Respir Viruses. 2015;9:85–93. 25557920 10.1111/irv.12302 PMC4353321

[25] World Health Organization. Pocket book of hospital care for children: Second edition: Guidelines for the management of common childhood illnesses. Available at: http://www.who.int/maternal_child_adolescent/documents/child_hospital_care/en. Accessed November 28, 2017. 24006557

[26] Scott JA Wonodi C Moisi JC. The definition of pneumonia, the assessment of severity, and clinical standardization in the Pneumonia Etiology Research for Child Health study. Clin Infect Dis. 2012;54(Suppl 2):S109–S116. 22403224 10.1093/cid/cir1065 PMC3297550

[27] Crawley J Prosperi C Baggett HC. Standardization of clinical assessment and sample collection across All PERCH Study Sites. Clin Infect Dis. 2017;64(Suppl 3):S228–S237. 28575355 10.1093/cid/cix077 PMC5447838

[28] Higdon MM Hammitt LL Deloria Knoll M. Should controls with respiratory symptoms be excluded from case-control studies of pneumonia etiology? Reflections from the PERCH Study. Clin Infect Dis. 2017;64(suppl 3):S205–S212. 28575354 10.1093/cid/cix076 PMC5447853

[29] World Health Organization. WHO AnthroPlus software 2009. Available at: http://www.who.int/growthref/tools/en. Accessed July 28, 2018.

[30] Cherian T Mulholland EK Carlin JB. Standardized interpretation of paediatric chest radiographs for the diagnosis of pneumonia in epidemiological studies. Bull World Health Organ. 2005;83:353–359. 15976876 PMC2626240

[31] Fancourt N Deloria Knoll M Barger-Kamate B. Standardized interpretation of chest radiographs in cases of pediatric pneumonia from the PERCH Study. Clin Infect Dis. 2017;64(suppl 3):S253–S261. 28575359 10.1093/cid/cix082 PMC5447844

[32] Murdoch DR O’Brien KL Driscoll AJ. Laboratory methods for determining pneumonia etiology in children. Clin Infect Dis. 2012;54(Suppl 2):S146–S152. 22403229 10.1093/cid/cir1073

[33] Feikin DR Fu W Park DE. Is higher viral load in the upper respiratory tract associated with severe pneumonia? Findings from the PERCH Study. Clin Infect Dis. 2017;64(suppl 3):S337–S346. 28575373 10.1093/cid/cix148 PMC5447843

[34] Park DE Baggett HC Howie SRC. Colonization density of the upper respiratory tract as a predictor of Pneumonia-*Haemophilus influenzae*, *Moraxella catarrhalis*, *Staphylococcus aureus*, and *Pneumocystis jirovecii*. Clin Infect Dis. 2017;64(suppl 3):S328–S336. 28575367 10.1093/cid/cix104 PMC5612712

[35] Baggett HC Watson NL Deloria Knoll M. Density of upper respiratory colonization with *Streptococcus pneumoniae* and its role in the diagnosis of pneumococcal pneumonia among children aged <5 years in the PERCH Study. Clin Infect Dis. 2017;64(suppl 3):S317–S327. 28575365 10.1093/cid/cix100 PMC5850437

[36] Morpeth SC Deloria Knoll M Scott JAG. Detection of Pneumococcal DNA in blood by polymerase chain reaction for diagnosing pneumococcal pneumonia in young children from low- and middle-income countries. Clin Infect Dis. 2017;64(suppl 3):S347–S356. 28575371 10.1093/cid/cix145 PMC5447841

[37] Deloria Knoll M Morpeth SC Scott JAG. Evaluation of pneumococcal load in blood by polymerase chain reaction for the diagnosis of pneumococcal pneumonia in young children in the PERCH Study. Clin Infect Dis. 2017;64(suppl 3):S357–S367. 28575374 10.1093/cid/cix149 PMC5447847

[38] Driscoll AJ Deloria Knoll M Hammitt LL. The effect of antibiotic exposure and specimen volume on the detection of bacterial pathogens in children with pneumonia. Clin Infect Dis. 2017;64(suppl 3):S368–S377. 28575366 10.1093/cid/cix101 PMC5447850

[39] Zhenke W. BAKER for the PIA analysis. Available at: http://zhenkewu.com. Accessed September 13, 2018.

[40] Deloria Knoll M Fu W Shi Q. Bayesian estimation of pneumonia etiology: epidemiologic considerations and applications to the Pneumonia Etiology Research for Child Health Study. Clin Infect Dis. 2017;64(suppl 3):S213–S227. 28575370 10.1093/cid/cix144 PMC5447849

[41] Wu Z Deloria-Knoll M Zeger SL. Nested partially latent class models for dependent binary data; estimating disease etiology. Biostatistics. 2017;18:200–213. 27549120 10.1093/biostatistics/kxw037

[42] Weber AM Areerat P Fischer JE. Factors associated with diagnostic evaluation for tuberculosis among adults hospitalized for clinical pneumonia in Thailand. Infect Control Hosp Epidemiol. 2008;29:648–657. 18564918 10.1086/588684

[43] Nicol MP Zar HJ. New specimens and laboratory diagnostics for childhood pulmonary TB: progress and prospects. Paediatr Respir Rev. 2011;12:16–21. 21172670 10.1016/j.prrv.2010.09.008 PMC3052970

[44] Onozaki I Law I Sismanidis C. National tuberculosis prevalence surveys in Asia, 1990-2012: an overview of results and lessons learned. Trop Med Int Health. 2015; 20:1128–1145. 25943163 10.1111/tmi.12534

[45] World Health Organization. Global Tuberculosis Report 2012. Available at: https://apps.who.int/iris/bitstream/handle/10665/75938/9789241564502_eng.pdf;jsessionid=E958BEFEA87CD9172509DA9D864F7FF5?sequence=1. Accessed May 17, 2019.

[46] Goto M Al-Hasan MN. Overall burden of bloodstream infection and nosocomial bloodstream infection in North America and Europe. Clin Microbiol Infect. 2013;19:501–509. 23473333 10.1111/1469-0691.12195

[47] Morgan OW Chittaganpitch M Clague B. Hospitalization due to human parainfluenza virus-associated lower respiratory tract illness in rural Thailand. Influenza Other Respir Viruses. 2013;7:280–285. 22716273 10.1111/j.1750-2659.2012.00393.x PMC5779843

[48] Prachayangprecha S Makkoch J Suwannakarn K. Epidemiology of seasonal influenza in Bangkok between 2009 and 2012. J Infect Dev Ctries. 2013;7:734–740. 24129626 10.3855/jidc.2929

[49] Prachayangprecha S Vichaiwattana P Korkong S. Influenza activity in Thailand and occurrence in different climates. Springerplus. 2015;4:356. 26191483 10.1186/s40064-015-1149-6 PMC4503703

[50] Ioannidis JP Worthington M Griffiths JK. Spectrum and significance of bacteremia due to *Moraxella catarrhalis*. Clin Infect Dis. 1995;21:390–397. 8562749 10.1093/clinids/21.2.390

[51] Abuhammour WM Abdel-Haq NM Asmar BI. *Moraxella catarrhalis* bacteremia: a 10-year experience. South Med J. 1999;92:1071–1074. 10586832 10.1097/00007611-199911000-00005

[52] Rhodes J Dejsirilert S Maloney SA. Pneumococcal bacteremia requiring hospitalization in rural Thailand: an update on incidence, clinical characteristics, serotype distribution, and antimicrobial susceptibility, 2005–2010. PLoS ONE. 2013;8:e66038. 23840395 10.1371/journal.pone.0066038 PMC3694083

[53] Rhodes J Hyder JA Peruski LF. Antibiotic use in Thailand: quantifying impact on blood culture yield and estimates of pneumococcal bacteremia incidence. Am J Trop Med Hyg. 2010;83:301–306. 20682872 10.4269/ajtmh.2010.09-0584 PMC2911175

[54] Chamany S Burapat C Wannachaiwong Y. Assessing the sensitivity of surveillance for pneumonia in rural Thailand. Southeast Asian J Trop Med Public Health. 2008;39:549–556. 18564697

[55] UNICEF. Thailand Statistics. 2010. Available at: https://www.unicef.org/infobycountry/Thailand_statistics.html#113. Accessed January 11, 2018.

