# Supplementary material for: Etiology and Clinical Characteristics of Severe Pneumonia Among Young Children in Thailand: Pneumonia Etiology Research for Child Health (PERCH) Case–Control Study Findings, 2012–2013
Source: Pediatr Infect Dis J. 2021 Aug 25;40(9):S91–S100. doi: 10.1097/INF.0000000000002768 (PMC8448397; doi:10.1097/INF.0000000000002768)
Supplement: Supplementary file 1 [file inf-40-s91-s001.docx]

**Supplemental Digital Content 1, Table. Cases that died within 30 days of discharge – PERCH, Thailand, 2012-2013**

| **Case**  **#** | **Pneumonia Severity** | **Age**  **(months)** | **Blood culture result** | **Chest radiograph result** | **Hypoxic^a^** | **Antibiotic pretreatment prior to specimen collection^b^** | **Premature** | **Died in hospital** | **Days in hospital** | **Comorbidity** |
| --- | --- | --- | --- | --- | --- | --- | --- | --- | --- | --- |
| 1 | Severe | 2 | Neg | Normal | No | Yes | No | No | 17 | None |
| 2 | Severe | 2 | *Pseudomonas aeroginosa* | Normal | Unk | Yes | No | Yes | 3 | None |
| 3 | Severe | 7 | Neg | Consolidation | No | No | No | No | 8 | Developmental delay, severe malnutrition |
| 4 | Severe | 12 | Neg | Consolidation + Other infiltrate | Yes | Yes | No | Yes | 7 | Developmental delay, heart disease |
| 5 | Severe | 45 | *Streptococcus pyogenes* | Consolidation | No | Yes | Yes | Yes | 5 | Developmental delay |
| 6 | Very Severe | 1 | Neg | Consolidation | Yes | Yes | No | No | 3 | Heart disease |
| 7 | Very severe | 3 | Neg | Consolidation | No | No | No | No | 7 | Developmental delay |
| 8 | Very severe | 5 | Neg | Normal | Yes | Yes | No | No | 8 | Developmental delay, heart disease |
| 9 | Very severe | 7 | Neg | Other infiltrate | Yes | No | No | No | 7 | Severe malnutrition, thalassemia |

^a^ Oxygen saturation <92% on room air.

^b^ Presence of antibiotics by serum, antibiotics at the referral hospital, clinician report of antibiotics prior to specimen collection or antibiotics prior to specimen collection based on time of specimen collection and time of antibiotic administration
