# Supplementary material for: Etiology and Clinical Characteristics of Severe Pneumonia Among Young Children in Thailand: Pneumonia Etiology Research for Child Health (PERCH) Case–Control Study Findings, 2012–2013
Source: Pediatr Infect Dis J. 2021 Aug 25;40(9):S91–S100. doi: 10.1097/INF.0000000000002768 (PMC8448397; doi:10.1097/INF.0000000000002768)
Supplement: Supplementary file 2 [file inf-40-s91-s002.docx]

**Supplemental Digital Content 2, Table. Co-detection of organisms by nasopharyngeal/ oropharyngeal PCR – PERCH, Thailand, 2012-2013**

|  | **All Cases N=223** | **CXR+ Cases N=98** | **All Controls N=657** | **All Cases vs All Controls** | **CXR+ Cases vs All Controls** |  |
| --- | --- | --- | --- | --- | --- | --- |
|  | n (%) | n (%) | n (%) | p-value | p-value) |  |
| Number of organisms, any positivity |  |  |  |  |  |  |
| Mean number of organisms, any positivity (SD) | 3.00 (1.41) | 3.02 (1.26) | 3.23 (1.46) |  |  |  |
| Median (IQR) | 3.0 (2.0, 4.0) | 3.0 (2.0, 4.0) | 3.0 (2.0, 4.0) |  |  |  |
| 0 | 8 (3.6) | 1 (1.0) | 19 (2.9) | .61 | .33 |  |
| 1 | 24 (10.8) | 9 (9.2) | 55 (8.4) |  |  |  |
| 2 | 47 (21.1) | 24 (24.5) | 115 (17.5) |  |  |  |
| 3 | 67 (30.0) | 33 (33.7) | 202 (30.7) |  |  |  |
| 4+ | 77 (34.5) | 31 (31.6) | 262 (39.9) |  |  |  |
| Pathogen patterns, any positivity |  |  |  |  |  |  |
| Bacteria only |  |  |  | .53 | .56 |  |
| Single bacteria | 10 (4.5) | 4 (4.1) | 31 (4.7) |  |  |  |
| 2 or more bacteria | 15 (6.7) | 5 (5.1) | 63 (9.6) |  |  |  |
| Virus only |  |  |  |  |  |  |
| Single virus | 14 (6.3) | 5 (5.1) | 24 (3.7) |  |  |  |
| 2 or more viruses | 10 (4.5) | 6 (6.1) | 23 (3.5) |  |  |  |
| Bacterial-Viral | 166 (74.4) | 77 (78.6) | 493 (75.0) |  |  |  |
|  | | | | | | |
