# Supplementary material for: Etiology and Clinical Characteristics of Severe Pneumonia Among Young Children in Thailand: Pneumonia Etiology Research for Child Health (PERCH) Case–Control Study Findings, 2012–2013
Source: Pediatr Infect Dis J. 2021 Aug 25;40(9):S91–S100. doi: 10.1097/INF.0000000000002768 (PMC8448397; doi:10.1097/INF.0000000000002768)
Supplement: Supplementary file 4 [file inf-40-s91-s004.docx]

**Supplemental Digital Content 4, Table. Etiologic fractions and 95% credible intervals, HIV-uninfected cases – PERCH, Thailand, 2012-2013**

|  | **Etiologic Fraction (Lower 95% Credible Interval^a^ – Upper 95% Credible Interval)** | |
| --- | --- | --- |
|  | **CXR+ Cases (n=98)** | **All Cases (n=223)** |
| **Bacteria** |  |  |
| *B. pertussis* | 0.27 (<0.01 - 2.03) | 0.13 (<0.01 – 0.93) |
| *C. pneumoniae* | 0.27 (<0.01 - 2.01) | 0.12 (<0.01 – 0.93 |
| *H. influenzae* (high density)^b^ | 3.46 (<0.01 - 12.90) | 5.70 (0.07 – 11.99) |
| Type b | 0.35 (<0.01 - 2.70) | 0.15 (<0.01 – 1.13) |
| Non-b | 3.11 (<0.01 - 12.51) | 5.55 (<0.01 – 11.79) |
| Legionella species | 0.26 (<0.01 - 1.95) | 0.12 (<0.01 - 0.93) |
| *M. pneumoniae* | 1.85 (<0.01 - 8.67) | 0.62 (<0.01 – 3.78) |
| *M. tuberculosis*^c^ | 10.37 (1.62- 25.56) | 5.46 (0.80 – 14.26) |
| *S. aureus* | 1.03 (<0.01 - 6.85) | 0.36 (<0.01 – 2.61) |
| *S. pneumoniae* (high density)^b^ | 0.74 (<0.01 - 3.87) | 0.30 (<0.01 – 1.63) |
| Salmonella species | 0.49 (<0.01 - 3.87) | 0.22 (<0.01 – 1.64) |
| Other bacteria, high sensitivity^d^ | 2.29 (<0.01 - 12.93) | 2.87 (0.11 – 9.94) |
| Other bacteria, low sensitivity^e^ | 20.23 (5.00 - 40.43) | 20.15 (7.48 -36.30) |
| **Fungi** |  |  |
| *Candida* species^f^ | 2.35 (<0.01 – 16.20) | 1.15 (<0.01 – 8.22) |
| *P. jirovecii* (high density)^b^ | 0.27 (<0.01 - 2.02) | 0.12 (<0.01 – 0.93) |
| **Viruses** |  |  |
| Adenovirus | 0.90 (<0.01 – 6.12) | 0.90 (<0.01 – 5.29) |
| Bocavirus | 1.36 (<0.01 – 8.98) | 0.65 (<0.01 – 4.66) |
| CMV (high density)^b^ | 0.55 (<0.01 – 4.19) | 0.33 (<0.01 – 2.55) |
| Coronavirus (43, 63, 229, HKU)^g^ | 1.65 (<0.01 – 6.92) | 1.34 (<0.01 – 4.76) |
| HMPV A/B | 2.13 (<0.01 – 8.07) | 2.11 (<0.01 – 5.97) |
| Influenza (A,B,C) ^g^ | 1.53 (0.01 – 5.60) | 2.46 (0.26 – 5.81) |
| Parainfluenza (1, 2, 3, 4) ^g^ | 3.00 (0.08 – 9.21) | 4.03 (0.73 – 8.71) |
| PV/EV | 1.29 (<0.01 – 7.34) | 4.68 (<0.01 – 11.11) |
| Rhinovirus | 4.49 (<0.01 – 15.72) | 16.08 (7.69 – 26.31) |
| RSV A/B | 34.56 (22.21 – 49.84) | 27.52 (19.26 – 37.98) |
| **Not otherwise specified** | 4.67 (<0.01 - 21.13) | 2.53 (<0.01 - 14.48) |
| **Summary Estimates** |  |  |
| Bacteria (excluding *M. tuberculosis)* | 30.63 (12.68 - 50.57) | 30.48 (16.93 – 45.80) |
| Viruses | 51.46 (34.27 - 69.86) | 60.13 (45.75 – 74.11) |

^a^ Bayesian analysis by design requires all pathogens to be included in the etiology pie, thus no pathogen can have a lower bound of 0%; a lower bound of <0.01% indicates there is insufficient evidence to support the pathogen as a definite cause of pneumonia (i.e., 0% is a possibility).

b High density defined by use of NP-OP PCR density thresholds for four pathogens: *Pneumocystis jirovecii*, 4 log10 copies per mL; *Haemophilus influenzae*, 5.9 log10 copies per mL; cytomegalovirus, 4.9 log10 copies per mL; *Streptococcus pneumoniae*, 6.9 log10 copies per mL

^c^ This etiologic fraction is based on two confirmed cases that were positive for *M. tuberculosis* on induced sputum culture.

^d^ Enterobacteriaceae and *Neisseria meningitidis*; high sensitivity = 10-50%.

^e^ *Moraxella catarrhalis*, Non-fermenting gram-negative rods, and other Streptococcus; low sensitivity=5-15%.

^f^ Includes *Candida species* and *Candida albicans*.

^g^ The etiology of each subtype was estimated separately and them subsequently summed together.
